# Adaptive Evolution of *Sporosarcina pasteurii* Enhances Saline–Alkali Resistance for High-Performance Concrete Crack Repair via MICP

**DOI:** 10.3390/microorganisms13071526

**Published:** 2025-06-30

**Authors:** Jieyu Liu, Huaihua Xu, Min Dong, Zilin Cheng, Chenkai Mi, Shuai Sun, Ruiying Zhu, Peipei Han

**Affiliations:** College of Bioengineering, Tianjin University of Science and Technology, Tianjin 300457, China; 18732971736@163.com (J.L.); xuhuaihua_3705@163.com (H.X.); dm13934788442@163.com (M.D.); czl907604018@163.com (Z.C.); 15110656974@163.com (C.M.); shuaioffice@163.com (S.S.); zry@tust.edu.cn (R.Z.)

**Keywords:** MICP, *Sporosarcina pasteurii*, laboratory adaptive evolution, genome, transcriptome, compressive strength, anti permeability

## Abstract

Microbially induced calcium carbonate precipitation (MICP) has emerged as a research focus in concrete crack remediation due to its environmental compatibility and efficient mineralization capacity. The hypersaline conditions of seawater (average 35 g/L NaCl) and alkaline environments (pH 12) within concrete cracks pose significant challenges to the survival of mineralization-capable microorganisms. To enhance microbial tolerance under these extreme conditions, this study employed a laboratory adaptive evolution strategy to successfully develop a *Sporosarcina pasteurii* strain demonstrating tolerance to 35 g/L NaCl and pH 12. Comparative analysis of growth characteristics (OD_600_), pH variation, urease activity, and specific urease activity revealed that the evolved strain maintained growth kinetics under harsh conditions comparable to the parental strain under normal conditions. Subsequent evaluations demonstrated the evolved strain’s superior salt–alkali tolerance through enhanced enzymatic activity, precipitation yield, particle size distribution, crystal morphology, and microstructure characterization under various saline–alkaline conditions. Whole-genome sequencing identified five non-synonymous mutated genes associated with ribosomal stability, transmembrane transport, and osmoprotectant synthesis. Transcriptomic profiling revealed 1082 deferentially expressed genes (543 upregulated, 539 downregulated), predominantly involved in ribosomal biogenesis, porphyrin metabolism, oxidative phosphorylation, tricarboxylic acid (TCA) cycle, and amino acid metabolism. In concrete remediation experiments, the evolved strain achieved superior performance with 89.3% compressive strength recovery and 48% reduction in water absorption rate. This study elucidates the molecular mechanisms underlying *S. pasteurii*’s salt–alkali tolerance and validates its potential application in the remediation of marine engineering.

## 1. Introduction

The durability of reinforced concrete (RC) structures in marine environments is significantly compromised, primarily caused by corrosion of steel reinforcement resulting from chloride ion intrusion [1]. Chloride ions penetrate the concrete and degrade the passivation layer of the reinforcement, thereby inducing corrosion [2]. The corrosion products of the reinforcement expand, exceeding twice their original volume, which leads to concrete cracking [3]. As the cracks propagate, the diffusion rate of chloride ions increases, thereby accelerating the corrosion process of the reinforcement. This cyclic degradation mechanism ultimately results in a significant reduction in the durability and load-carrying capacity of RC structures [4,5]. Therefore, sealing concrete cracks and mitigating the diffusion rate of chloride ions are essential for prolonging the service life of RC structures. Currently, commonly employed methods for repairing concrete cracks include organic-coated surface treatments and grouting with cementation materials or mortar [6]. However, these conventional methods are not only costly but also contribute to environmental pollution through the release of volatile gases generated during their production [7]. Furthermore, both economic costs and environmental characteristics of materials must be critically evaluated when selecting repair materials for concrete cracks.

In nature, bacteria induce calcium carbonate precipitation by facilitating metabolic processes and secreting enzymes, a phenomenon academically termed microbially induced calcium carbonate precipitation (MICP) [8]. Since Boquet et al. [9] first documented this process, MICP technology has garnered significant attention from researchers globally. Studies have isolated diverse bacteria capable of calcium carbonate precipitation from natural environments and enhanced calcium carbonate production yields through optimized culture conditions. Under laboratory conditions, MICP technology has demonstrated efficacy in enhancing the compressive strength and durability of repaired specimens. For instance, Sun et al. [10] achieved a 21.2% increase in compressive strength compared to unrepaired specimens by applying bacterial solutions via dropwise inoculation. Similarly, Chunxiang Qian et al. [11] reduced the permeability coefficient of concrete specimens by 86.1% using spray-based application of bacterial solutions. However, the marine environment’s high salinity (35 g/L) and the alkaline conditions (pH = 12) of concrete crevice fluids significantly inhibit microbial survival, enzymatic activity, and calcium carbonate precipitation, substantially restricting the practical applicability of microbial mineralization. The high pH value in cement-based materials may hinder microbial-induced calcium precipitation during biomineralization processes. The impact of extreme temperatures (55 °C) and pH (13.6) on viability and urea hydrolysis was greatest, but significantly reduced with exposure to milder temperatures (45 °C) and pH (12.9); the optimal conditions for urease activity are 30 °C and pH 9 [12,13,14]. Current research on the influence of marine environmental conditions on MICP efficacy remains limited, particularly regarding crack repair in marine concrete structures, and no effective method exists to introduce bacteria into cracks in real-world marine infrastructure [15,16]. To address these challenges, further investigation is required to develop methodologies for effective bacterial introduction into cracks while mitigating the inhibitory effects of high-salinity (35 g/L) marine environments and alkaline (pH = 12) crevice fluids on microbial activity.

To enhance strain adaptation to the high-salinity and alkaline conditions of marine concrete crevices, adaptive laboratory evolution (ALE) was employed for strain enhancement. ALE selects for high-performance strains through prolonged incubation in controlled growth environments or under selective pressure [17]. For instance, Wang et al. [18] isolated seawater-adapted strains via gradient-evolution of *S. pasteurii* in artificial seawater, while Vos et al. [19] developed low-temperature-evolved *Bacillus megaterium* strains with elevated calcium carbonate yields. Previous studies on microbial mineralization strains have not comprehensively addressed evolutionary processes under high-salinity conditions. Therefore, this study aims to investigate adaptive evolution in high-salinity and alkaline environments to develop strains with enhanced tolerance to such extreme conditions.

Transcriptomic technologies enable comprehensive monitoring of gene expression changes in organisms under varying conditions, elucidating regulatory mechanisms and predicting unknown gene functions [20,21]. For example, Sah et al. [22] identified novel genes and pathways linked to eggshell biomineralization via transcriptome sequencing of chicken uterine tissue. Similarly, Michiko et al. [23] detected a high abundance of mitochondrial respiratory chain protein transcripts in the mineralized tooth regions of stone turtles, identifying genes critical to tooth biomineralization. Gupta et al. [24] reported that the adaptive evolution of Lactococcus lactis under isobutanol stress resulted in marked alterations in membrane transport and metabolism-related gene expression. Conversely, genome resequencing elucidates non-coding sequence functionality by analyzing genomic variations (e.g., SNPs and indels), offering novel insights into strain metabolic regulation [25]. Wang et al. [26] employed macrogenome sequencing to identify functional genes associated with organic carbon mineralization in recalcitrant carbon aggregates. Xu et al. [27] demonstrated that the adaptive evolution of Fusobacterium lutetium for phenol degradation involved mutations in detoxification enzymes and resistance genes, while Elsayed et al. [28] linked PP_3350 and ttgB gene mutations in Pseudomonas malodorata to coumaric acid tolerance. In this study, transcriptomic and genomic resequencing analyses of *S. pasteurii* under diverse environmental conditions revealed mechanisms underlying gene expression and genomic variation, offering critical insights into its biological functions and adaptive evolution.

Marine concrete structures globally face durability and safety challenges due to cracking. When cracks appear in port concrete, if the cracks are not repaired in time, they may lead to gradual changes in the structure of the concrete, thus seriously affecting its strength [29]; this further leads to the formation of a transmission channel for the entry of water and other harmful media into the interior of the concrete, which can cause a decrease in the durability of the concrete and affect the life of the concrete [30]. Crack repair will prevent water and harmful agents from entering the interior of the concrete, and it takes more time to penetrate into the specimen; thus, the lower the water absorption in the test results, the better the specimen’s impermeability. Microbial mineralization technology offers an environmentally sustainable repair method that utilizes calcium carbonate deposition to seal microcracks. However, its application is constrained by the high-salinity and alkaline conditions typical of marine environments.

In this study, we isolated *S. pasteurii* strains capable of thriving under such extreme conditions using laboratory adaptive evolution strategies. Through transcriptomic and genomic analyses, we characterized the morphological features, physiological adaptations, and calcium carbonate production efficiency of these strains, establishing a scientific foundation for advancing microbial mineralization technology in marine concrete repair.

## 2. Material and Method

### 2.1. Strain and Culture Method

The experimental strain used was *S. pasteurii* (derived from ARTP-induced mutant strains, named B11) [31]. The activation medium for *S. pasteurii* consisted of a NH_4_-YE medium containing yeast extract (20 g/L), (NH_4_)_2_SO_4_ (10 g/L), and 0.13 mol/L Tris-HCl buffer (pH 9.0). Solid media contained 20 g/L agar. The parent strain medium for *S. pasteurii* contained yeast extract (20 g/L), (NH_4_)_2_SO_4_ (10 g/L), 0.13 mol/L Tris buffer, and 20 g/L agar. The evolution medium for *S. pasteurii* included yeast extract (20 g/L), urea (10 g/L), and 0.13 mol/L Tris buffer. For adaptive evolution, NaCl was added to the medium to achieve target salinity levels. Media were sterilized at 121 °C for 20 min, and pH was adjusted using NaOH to the corresponding bacterial tolerance value during the evolution process. Single colonies of *S. pasteurii* were inoculated into test tubes and incubated at 30 °C with 200 rpm agitation for 24 h until the stationary phase was reached. All chemicals and reagents were analytical-grade. Ultrapure water was prepared using a Milli-Q^®^ water purification system (Millipore, Bedford, MA, USA).

### 2.2. Laboratory Adaptive Evolutionary Approaches

ASTM D1141-98 (2013) [32] specifies that seawater alternatives contain an average salinity of 35 g/L, with NaCl representing the component of highest concentration. Thus, NaCl was selected as the primary agent for salt-tolerance adaptation. Additionally, to simulate the highly alkaline conditions of concrete crevice fluids (pH = 12), NaOH was incorporated into the experimental design. For this study, triplicate experimental setups were prepared using 250 mL conical flasks, each containing 50 mL of culture medium.

**Figure 1:**
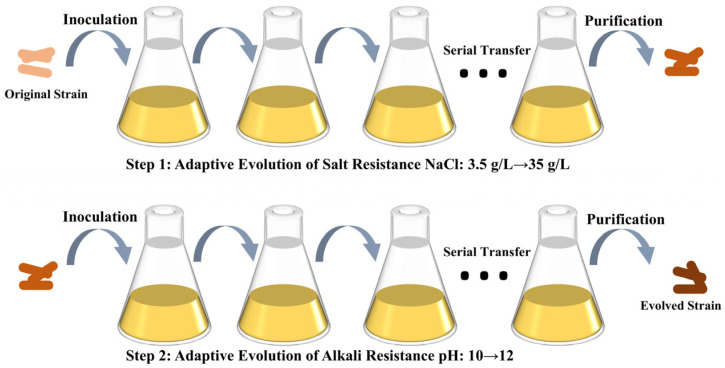
Adaptive evolutionary approach to the laboratory.

Urease-producing *S. pasteurii* was inoculated at 5% (*v*/*v*) into salt-tolerant evolution medium. Salt stress tolerance was assessed using salinity gradients of 0, 1.75, 3.5, 7, 14, 21, 28, and 35 g/L. For alkali tolerance evaluation, the bacterium was inoculated at 5% (*v*/*v*) into medium with pH gradients (8.0–12.0) adjusted using NaOH to evaluate alkaline stress tolerance. Based on preliminary tolerance assessments, *S. pasteurii* was sequentially transferred to a salt-containing medium. The preliminary tolerance of *S. pasteurii* was evaluated based on the average tolerance of the original strain under different stress environments (different salinity, pH). At each stage, 5% (*v*/*v*) of the bacterial inoculum was passaged until it reached a stable growth stage, and then the next generation of adaptive evolution was carried out. After salt tolerance evaluation, the 35 g/L NaCl-adapted strain was transferred to a basic pH medium and cultured until the bacterial cells reached a stable phase. The pH was incrementally increased, with cultures incubated until stabilization at each step, until the strain acclimated to 35 g/L NaCl and pH 12. The adaptive evolution process of *Bacillus subtilis* is shown in Figure 1. Control strains were inoculated into a saline stress-free medium and incubated under identical conditions.

### 2.3. Measurement of Urease Activity

Urease activity was quantified via the conductivity method, as described by Whiffin [32], wherein conductivity change rates exhibit a linear correlation with enzymatic activity. Thus, enzymatic activity was defined by urea hydrolysis rates, with higher hydrolysis corresponding to greater urease activity. Conductivity changes were measured over a 5 min interval to quantify this relationship.

(1) $$Urease\;activity\;(mM/min)=\frac{Conductivity\;change\;value}{5}\times 11.11\times 10$$

### 2.4. Measurement of Mineralization Product

Fermentation broth from pre- and post-evolution cultures of *S. pasteurii* was mixed with a nutrient salt solution containing dissolved calcium acetate and urea. The mineralization reaction was incubated at 30 °C for 24 h under static conditions. The precipitate was collected, dried, and weighed to quantify mineralization yield. Simultaneously, Ca^2+^ utilization was calculated based on supernatant calcium depletion.

The mineralization yield and calcium utilization efficiency were calculated using the following relationships:

m(CaCO_3_): Dry weight of calcium carbonate precipitate (g);

Cₑ: Concentration of the added calcium source (mol/L);

Mₑ: Molecular mass of the calcium source (g/mol);

Vₑ: Volume of the calcium source solution (L).

(2) $${Ca}^{2+}\;Utilization\;rate=\frac{Output}{Added}=\frac{m(CaCO_{3})\times \frac{40}{100}}{C_{E}\times M_{E}\times V_{E}\times \frac{40}{M_{E}}}=\frac{m(CaCO_{3})}{100\times C_{e}\times V_{e}}$$

### 2.5. Particle Size Analysis of Mineralized Sediment

Grind the collected sediment in a mortar to fully disperse the particles but avoid excessive fragmentation, ensuring the particle size distribution reflects the true state. Use the “Bettersize3000” laser particle size analyzer (Dandong, China) to analyze the particle size distribution of the sample. After turning on the pump and ultrasonic vibration instrument for background measurement, transfer the ground sample powder to a sample cell with an analysis range of 0.3–2000 μm. Collect data and then analyze the particle size distribution.

### 2.6. X-Ray Diffraction (XRD) Analysis

The calcium carbonate product was ground to 320-mesh particle size, and a minimum of 3 g was collected for analysis. The powder was pressed into the sample frame’s groove, ensuring the compacted material protruded slightly above the frame surface. A carrier sheet was gently pressed onto the surface, and excess powder was scraped to align the sample plane with the frame. The sample was mounted in the X-ray diffractometer (XRD) for analysis using Cu Kα radiation, with a scanning range of 2θ = 5–90°, a step size of 0.02°, and a scan rate of 2°/min. Data were exported to Jade 6 software for qualitative and quantitative phase analysis.

### 2.7. Scanning Electron Microscope Test (SEM) Analysis

Grind the calcium carbonate product obtained; cut off the appropriate size of the conductive adhesive, fixed to the scanning electron microscope sample stage; take a small amount of sample with a toothpick and fix it to the conductive adhesive, taking care that the samples do not contaminate each other; spray gold on the sample; bleed off the gas, place the samples, and observe on the machine.

### 2.8. Genome Resequencing

The whole genomes of the evolved strain (ES) and orginal strain (OS) of *B*. *pasteurii* were resequenced. For clarity, the evolved strain group was designated as ES, with its three biological replicates labeled ES1, ES2, and ES3; similarly, the orginal strain group was designated as OS, with replicates OS1, OS2, and OS3. These resequenced genomes were then compared to the reference genome. For consistency, the reference genome sequence of *S. pasteurii* was used for comparative analysis, *S. pasteurii*’s reference genome can be found in Supplementary Document S1.

DNA samples were assessed for quality prior to library construction, and raw sequencing data underwent quality control to generate clean reads. These reads were aligned to the reference genome and analyzed to identify single-nucleotide polymorphisms (SNPs). Protein physicochemical properties were predicted using the Expasy portal (SIB Swiss Institute of Bioinformatics, Lausanne, Switzerland). Transmembrane domains were identified using TMHMM 2.0 (Technical University of Denmark, Lyngby, Denmark), while secondary structures were predicted with SOPMA (Institute of Biology and Chemistry of Proteins, Lyon, France). Homology modeling was performed using SWISS-MODEL (https://swissmodel.expasy.org/, accessed on 14 April 2024), and structural visualization was conducted with PyMOL 2.4.0a0.

### 2.9. Transcriptome Sequencing 

RNA was extracted from *S. pasteurii* during the logarithmic growth phase, pre- and post-evolution. Cells were harvested via centrifugation (4000× *g*, 4 °C, 10 min), washed twice with phosphate-buffered saline (PBS), and resuspended. Total RNA was isolated using the TRIzol^®^ method. RNA integrity was assessed via agarose gel electrophoresis and spectrophotometry (A260/A280 ratio). A sequencing library was prepared using the NEBNext^®^ Ultra™ RNA Library Prep Kit and validated for quality. Qualified libraries were subjected to paired-end sequencing (2 × 150 bp) on an Illumina NovaSeq 6000 platform. The reference genome used for comparison is shown in Document S1.

Differential gene analysis was performed using DEGseq2 1.48.1 with a threshold of significant difference of |log2FoldChange| ≥ 0 and DESeq2 padj ≤ 0.05 to identify differentially expressed genes; KEGG and GO databases of differentially expressed genes were functionally classified and enriched.

To validate the RNA-Seq results, five genes exhibiting high differential expression fold changes in these results were selected for qRT-PCR analysis. Reverse transcription of *S. pasteurii* mRNA was conducted using the Takara PrimeScript FAST RT Reagent Kit, alongside the gDNA Eraser Reverse Transcription Kit. RNA purity and concentration were assessed (OD_260_/OD_280_ = 1.8–2.2; effective concentration ≥ 5 nmol/L) and quantified using the Reverse Transcription Kit and the qPCR Kit. qPCR experiments were conducted using the Bestar SYBR Green qPCR Mastermix Kit (DBI Bioscience, Shanghai, China). Data analysis was performed using the 2^−ΔΔCT^ method, with 16S rRNA serving as an internal reference to normalize quantitative data. Primer sequences are presented in Table 1.

### 2.10. Concrete Crack Repair Test

Precast concrete specimens were prepared with 0.3 mm wide cracks using a water–cement ratio of 0.39 and a cement–sand ratio of 0.6. During casting, 100 mm × 100 mm × 100 mm concrete specimens were fabricated by inserting steel plates of predetermined thickness into molds to introduce artificial microcracks measuring 900 mm × 0.3 mm × 20 mm (length × width × depth).

Bacterial solutions of *S. pasteurii* (pre- and post-evolution) were cultured for 36 h, harvested, and stored at 4 °C for subsequent use. A nutrient medium containing 1.5 M calcium acetate and 1.5 M urea was prepared, mixed thoroughly, and stored at room temperature.

The preserved bacterial solution was thawed in a 30 °C water bath for 1 h prior to use. Using a sterile syringe, 5 mL of the nutrient medium was injected into the cracks, followed by 5 mL of the bacterial solution, initiating calcium carbonate precipitation. The cracks were repaired three times a day, with an interval of 8 h between each repair, for a total of six times. Complete crack filling was achieved within 48 h through calcium carbonate precipitation.

### 2.11. Evaluation of Restoration Effect

Water absorption testing was employed to evaluate crack repair efficacy, with lower water absorption indicating superior restoration. M_1_ (initial mass) was recorded after sealing areas other than cracks with wax to prevent water ingress through non-repaired regions. Specimens were inverted to submerge the repaired surface in water for predetermined intervals, then removed, gently blotted to remove surface moisture, and reweighed to determine M_2_ (post-immersion mass). The water absorption rate (E, %) was calculated as follows:

(3) $$E=[(M_{2}-M_{1})/M_{1}\times 100\%]$$

Compressive strength was measured to assess concrete integrity post-repair, following JTS/T 236-2019 standards [33]. Specimens were categorized into three groups: blank group (cracked, unrepaired), control group (cracked, repaired with original strains), and experimental group (cracked, repaired with evolved strains). Triplicate specimens were tested for each group. The mean value was reported unless the maximum or minimum deviation exceeded 15%, in which case the median value was used.

### 2.12. Statistical Analysis

To ensure reproducibility of the results, each treatment group was cultured three times; the values are expressed as the mean ± standard deviation. The data were analyzed through the independent samples *t*-test using the SPSS statistical software (version 20.0).

## 3. Results and Discussion

### 3.1. Salt and Alkali Stress Tolerance of S. pasteurii

**Figure 2:**
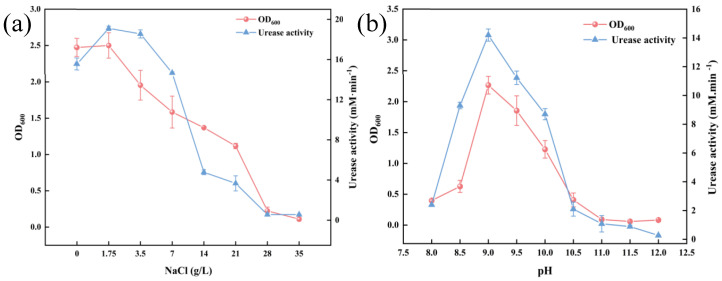
Salt stress tolerance results (**a**) and alkali stress tolerance results (**b**) of *S. pasteurii.*

Previous studies indicate that the optimal pH for microbial-induced calcium carbonate precipitation (MICP)-capable strains is approximately 8, aligning with the urease activity optimum range of pH 7–8, while systematic evaluations of salinity tolerance remain limited [34,35]. As shown in Figure 2a, the OD_600_ values and urease activity of *S. pasteurii* were assessed across increasing NaCl concentrations (*w*/*v*). Both parameters peaked at 1.75 g/L NaCl, beyond which a gradual decline occurred. To evaluate the strain’s adaptability to elevated salinity, 3.5 g/L NaCl was selected as the baseline salinity for subsequent salt stress evolution experiments. Similarly, Figure 2b illustrates the strain’s response to varying pH gradients. Maximum OD_600_ and urease activity were observed at pH 9. In order to challenge the alkaline tolerance of the strain and ensure its viability, a pH of 9.5 was chosen as the starting point for the alkaline stress evolution experiment.

### 3.2. ALE of S. pasteurii for Enhanced Saline Tolerance

To investigate adaptive evolution in *S. pasteurii*, we first focused on improving its salt stress tolerance. Successful adaptation to high-salinity environments provides a foundation for subsequent studies on alkali stress resilience under saline conditions. This sequential stress escalation enables systematic analysis of adaptive mechanisms and establishes a framework for engineering strains with enhanced environmental versatility. The original strain was then transferred to a culture medium without salt–alkali stress and cultured simultaneously as a control.

**Figure 3:**
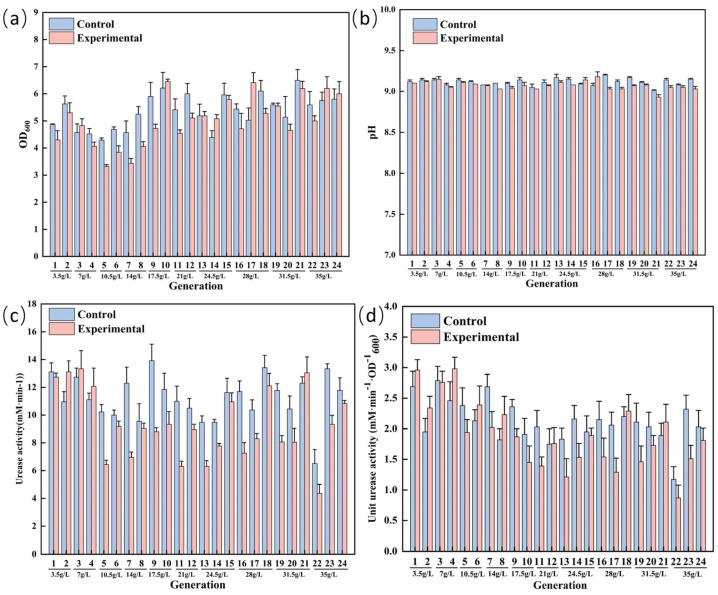
Laboratory adaptive evolution process of salt-stressed *S. pasteurii* OD_600_ (**a**), pH (**b**), urease activity (**c**), unit urease activity (**d**).

As shown in Figure 3, OD_600_ and urease activity were monitored as key phenotypic indicators during evolutionary passaging. Subculturing proceeded to the next generation only when no statistically significant differences (*p* > 0.05) in these parameters were observed between evolved and control groups. This approach aligns with established adaptive evolution strategies, such as those detailed by Katsuya et al. [36], who enhanced Aeromonas sp. growth under 0.225 M NaCl, and Li et al. [37], who evolved yeast strains tolerating 20% (*w*/*w*) NaCl. Notably, our evolved *S. pasteurii* strain maintained robust growth (OD_600_) and urease activity under extreme salinity (35 g/L NaCl), demonstrating successful adaptation to high-salinity conditions while retaining biomineralization capacity.

### 3.3. ALE of S. pasteurii for Enhanced Alkaline Tolerance

**Figure 4:**
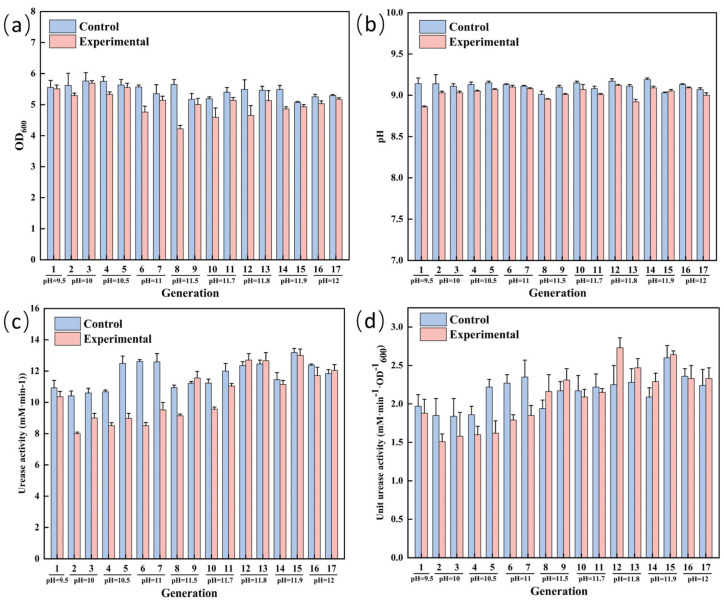
Laboratory adaptive evolution process of salt-tolerant *S. pasteurii* under alkali stress OD_600_ (**a**), pH (**b**), urease activity (**c**), unit urease activity (**d**).

Once *S. pasteurii* demonstrated stable growth and urease production at 35 g/L NaCl, adaptive evolution under alkaline stress (pH 12) was initiated. OD_600_ and urease activity served as primary phenotypic indicators, with subculturing performed only when no statistically significant differences (*p* > 0.05) in these parameters were observed between evolved and control groups. As illustrated in Figure 4, the evolved strain retained robust growth and urease activity under combined stress (35 g/L NaCl, pH 12), confirming successful adaptation to extreme saline–alkaline conditions. This approach aligns with established evolutionary strategies, such as those detailed by Li et al. [38], who enhanced Lactobacillus lactis acid tolerance (pH 4.6) via adaptive evolution, achieving a 43% increase in L-lactic acid production. In our study, the evolved *S. pasteurii* strain maintained stable urease expression and metabolic activity despite dual stressors, demonstrating its potential for biomineralization applications in harsh environments.

### 3.4. Comparison of Physiological Indexes of S. pasteurii

**Figure 5:**
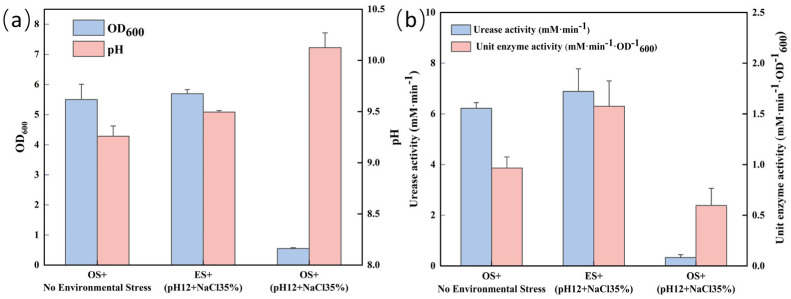
Comparison of OD_600_ and pH (**a**), urease activity and unit urease activity (**b**) between the original strain (OS) and the evolved strain (ES) of *S. pasteurii* under stress-free and stress conditions (pH = 12, NaCl 35 g/L).

Figure 5a shows that the OD_600_ value of the adaptively evolved *S. pasteurii* strain at pH 12 and NaCl concentration of 35 g/L far exceeded the performance of the original strain under the same stress condition, similar to the level of the original strain cultured under non-coercive conditions, which indicated that the growth ability of the evolved strain was significantly enhanced under high saline and alkaline environments. The pH value of the evolved strain cultured in a stress environment is similar to that of the original strain cultured in a non-stress environment. During the exponential growth process, the bacterial cells produce a large amount of organic acids, causing the pH of the culture medium to rapidly decrease [39,40]. In addition, due to the charge balance of ions in the system, the final pH value is almost always between 9 and 10 [14,41]. The original strain cannot grow in a stressful environment, and the pH value of the original strain after cultivation in a stressful environment is higher than the other two groups of experiments.

Figure 5b reveals that the evolved strains not only exhibited higher urease activity than the original strains under the same high saline conditions, but also excelled in unit urease activity. This suggests that the evolved strains have not only enhanced survivability in harsh environments, but are also more efficient in urease production. Collectively, these findings underscore the evolved strain’s dual advantage of enhanced survivability and biomineralization potential under extreme environmental challenges, supporting its applicability in biotechnology sectors requiring robust microbial performance in harsh conditions.

### 3.5. Comparison of Biomineralization Performance and Microstructural Characterization Analysis of S. pasteurii

**Figure 6:**
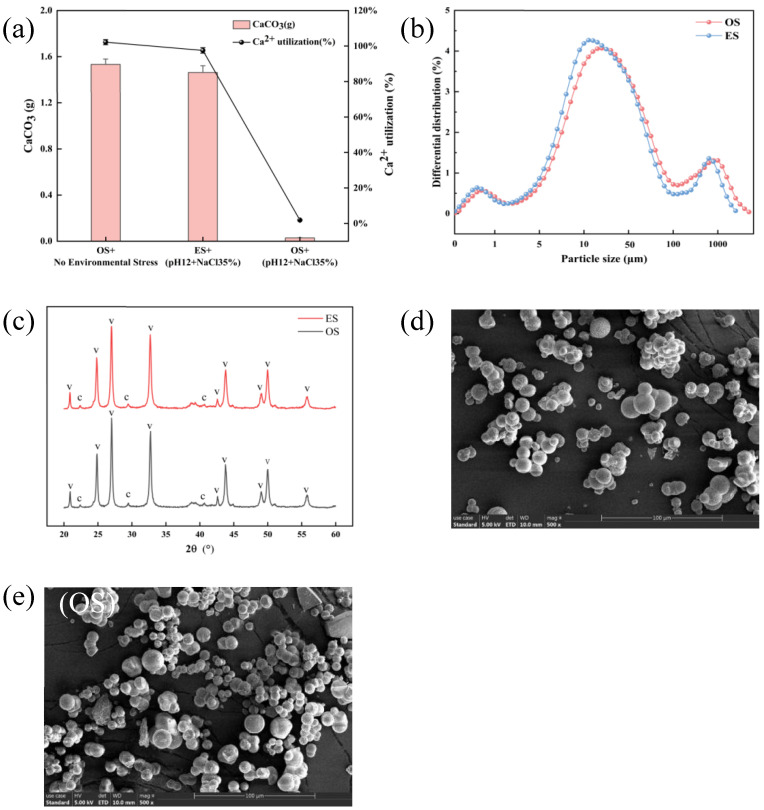
The precipitation amount of mineralized products and the utilization rate of calcium ions (**a**), the particle size of the precipitate (**b**), the XRD (c: the crystal form of calcium carbonate is calcite, v: the crystal form of calcium carbonate is vaterite) of the precipitate (**c**), the SEM of the precipitate produced by the mineralization reaction of the evolved strain (**d**), and the SEM of the precipitate produced by the mineralization reaction of the original strain (**e**).

It has been reported that the calcium carbonate production of *S. pasteurii* (ATCC 11859) is highest at pH 9, and its production decreases with increasing pH; the optimum pH for urease activity is at 7–8, and its activity also decreases with increasing pH [42,43,44]. Figure 6a shows that when the evolved strains cultured under simulated saline and alkaline stress conditions were compared with the original strains under non-stress conditions as well as under saline and alkaline stress, the following results can be obtained: the calcium carbonate production of the evolved strains cultured under saline and alkaline stress environments was comparable to that of the original strains cultured under non-stress environments, which indicates that the evolved strains, even under unfavorable environmental conditions, maintained a high level of calcium carbonate production. In contrast, the original strains in saline-stressed environments produced significantly lower amounts of calcium carbonate than the evolved strains, a difference that highlights the significant improvements gained by the evolved strains in the process of adaptive evolution. These results suggest that the evolved strains were not only able to maintain their growth and urease production capacity under saline and alkaline stress, but were also able to maintain similar levels of calcium carbonate production as under non-stress conditions. Such robustness positions these strains as promising candidates for applications requiring sustained microbial mineral precipitation in harsh settings, including saline–alkaline soil stabilization and concrete self-healing.

As shown in Figure 6b, particle size distributions of calcium carbonate differed markedly between original and evolved strains. original strain precipitates exhibited a broad size range (2.25–215.6 µm), with 84.45% of particles concentrated between 2.25 and 215.6 µm and a peak diameter of 21.24 µm. In contrast, evolved strain precipitates displayed a narrower distribution (1.54–114.4 µm), retaining 84.45% within this range but with a reduced peak diameter of 17.14 µm. This shift toward smaller particle sizes in the evolved strain suggests enhanced nucleation control, which may improve cementation density and mechanical strength in biomineralized materials.

When a high concentration of calcium acetate is used as the calcium source, the crystalline form of calcium carbonate will be dominated by spherulitic aragonite with a small amount of calcite [45,46]. The XRD analysis of Figure 6c shows that the calcium carbonate products of both the original and evolved strains contain diffraction peaks characteristic of both mineral phases, spherulite and calcite. Quantitative analysis by JADE software showed that the calcium carbonate product of the original strain contained 98.7% of spherical aragonite and 1.3% of calcite, whereas the calcium carbonate product of the evolved strain contained 97.1% of spherical aragonite and 2.9% of calcite, which is similar to the reported results. This indicates a slight increase in the proportion of calcite phase in the calcium carbonate produced by the evolved strain. The morphology of the calcium carbonate products was further confirmed by the SEM images in Figure 6d,e, which showed that most of the calcium carbonate particles were spherical in shape, which is in agreement with the results of XRD analysis. This spherical morphology may help to improve the cementation properties and application of calcium carbonate. Collectively, the evolved strains demonstrated optimized control over particle size distribution, polymorph selectivity, and morphological uniformity—advancements that enhance the functional performance of microbially induced calcium carbonate in engineering and environmental contexts.

### 3.6. Genome Sequencing Analysis

The results of the reference genome comparison are normal and can be used for subsequent variant detection and related analysis. The summary of sequencing data quality is shown in Table S1. The results of sequencing depth and coverage statistics are shown in Table S2.

Comparative genomic analysis of the evolved strain (ES) and original strain (OS) identified 10 mutations, including 5 non-synonymous mutations after excluding synonymous variants (Table 2). These included rRNA pseudouridine synthase, GntP family permeases, glycosyltransferases, and BCCT family transporters. Additionally, a phenylalanine-to-tyrosine substitution was detected within 1 kb flanking regions of the urease γ-subunit (ureC) and imidazole/amide deaminase genes.

The genes with non-synonymous mutations E2C16_02500, E2C16_10645, E2C16_11325, E2C16_12620 were predicted for their physicochemical properties using bioinformatics methods.

The predicted protein structure encoded by gene-encoding rRNA pseudouridine synthase (E2C16_02500) is shown in Figure S1. A non-synonymous Ala→Val substitution occurred in the 230-residue sequence, increasing the molecular weight from 26,083.96 Da to 26,112.01 Da without altering pI (5.86). The stable acidic protein (instability index: 30.17) exhibited minimal structural changes: the alpha helix increased from 30% to 30.87%, and the extended strand decreased from 25.2% to 22.17%, while GRAVY shifted from −0.385 to −0.375, indicating enhanced hydrophilicity. The mutant retained 99.57% identity to *S. pasteurii* wild-type pseudouridine synthase. The introduced methyl groups may affect substrate binding or enzymatic flexibility. Pseudouridylation defects impair ribosome biogenesis in *C. albicans* [47] and *E. coli* [48]; this mutation likely enhances ribosome stability under saline stress via altered conformational dynamics.

The predicted protein structure encoded by gene-encoding a GntP family permease (E2C16_10645) is shown in Figure S2. A Leu→Phe substitution in the 427-residue sequence increased molecular weight (45,069.78→45,103.80 Da) with unchanged pI (8.99). The stable basic transmembrane protein (7 predicted domains) showed reduced hydrophobicity (GRAVY: 0.865→0.853) and secondary structure shifts: the alpha helix increased (52.93%→54.57%), and the beta turn decreased (8.20%→7.73%). The mutation introduced a benzene ring, potentially altering transmembrane domain packing. Sharing 72.94% identity with *B. marinus* citrate transporters, this variant may enhance fructuronate transport in *S. pasteurii*, modulating pH homeostasis under saline–alkaline stress [49].

The predicted protein structure encoded by gene-encoding glycosyltransferase (E2C16_11325) is shown in Figure S3. An Asp→Tyr substitution in the 494-residue protein increased pI (7.56→7.97) and molecular weight (57,832.69→57,880.78 Da). The stable neutral hydrophilic protein (GRAVY: −0.607→−0.612) showed minor secondary structure adjustments: the alpha helix decreased (38.06%→36.23%), and the extended strand increased (21.26%→22.47%). The introduced phenolic hydroxyl group altered hydrophobicity. Structural homology with *S. pasteurii* glycosyltransferase was retained. Such mutations correlate with membrane integrity changes in *B. subtilis* (vancomycin resistance) [50] and virulence modulation in *P. gingivalis* [51], suggesting potential roles in salinity adaptation.

The predicted protein structure encoded by gene-encoding a BCCT family transporter protein (E2C16_12620) is shown in Figure S4. A Met→Leu substitution reduced molecular weight (59,347.71→59,329.67 Da) with unchanged pI (8.56). The alkaline-stable transmembrane protein (12 domains) exhibited increased hydrophobicity (GRAVY: 0.718→0.722) and structural shifts: the alpha helix rose (44.55%→47.56%), and the random coil decreased (29.7%→27.26%). Sharing 70.13% homology with *H. salina* choline transporters, the sulfur-to-methyl substitution may enhance glycine betaine transport. BCCT transporters mediate osmoprotectant uptake in *A. baumannii* [52,53] and *E. coli* [54], supporting osmotic stress adaptation in high-salinity environments.

### 3.7. Transcriptome Sequencing Analysis

Sequencing results of transcriptome analysis are presented in Table S3. As shown in Table S3, all samples from both the evolved strain (ES) and original strain (OS) groups exhibited Q20 scores > 97% with an error rate of merely 0.01%, demonstrating reliable sequencing quality suitable for subsequent analyses. Five genes were selected for qRT-PCR validation to assess the credibility of transcriptomic data. Validation results (Figure S5) revealed that the 2^−ΔΔCt^ value trends aligned with transcriptomic profiles, validating the reliability of transcriptomic data.

Using DESeq2 with screening thresholds (padj ≤ 0.05 and |log2FoldChange| ≥ 0), a total of 1082 differentially expressed genes (DEGs) were identified, including 543 upregulated and 539 downregulated genes. Notably, the salt–alkali-tolerant *B. pasteurii* evolutionary group exhibited a slightly higher proportion of upregulated genes compared to the control group, indicating distinct regulatory divergence. A volcano plot of DEGs is shown in Figure S6, where upregulated/downregulated genes and their statistical significance are visually represented. GO enrichment analysis (Figure S7) highlighted five predominant biological processes: the cellular protein metabolic process, translation, peptide metabolic process, polypeptide biosynthetic process, and cellular metabolic process. Cellular component terms were enriched in the intracellular organelle, non-membrane-bounded organelle, ribonucleoprotein complex, cytoplasmic region, and the ribosome. Molecular functions primarily involved structural constituents of the ribosome, molecular structure activity, electron transfer activity, and heme binding. KEGG pathway analysis (Figure S8) revealed that DEGs between the evolved strain (ES) and original strain (OS) were predominantly associated with the ribosome, porphyrin metabolism, oxidative phosphorylation, and tricarboxylic acid (TCA) cycle.

Ribosomal translation function was enhanced through the upregulation of elongation factor genes EF-Tu/EF-G to improve translational efficiency [55,56,57], which coordinated with the activation of the SecY protein channel genes to confer stress resistance [58]. Differential regulation was observed in the initiation factors: IF1 and IF3 promoted translational fidelity, while the downregulation of IF2 indicated impaired ribosome maturation [59,60,61]. The upregulation of rpL17 (RpoA subunit gene) maintained intracellular pH homeostasis via Na+/H+ antiport, whereas the downregulation of release factor RF1-associated genes (*rpL31*/*rpL3*) led to peptide chain release inhibition. These findings suggest, to some extent, a compromised release of nascent polypeptide chains from the ES under saline–alkali stress [62]. Differentially expressed genes in ribosomal metabolic pathways are listed in Table S4.

**Figure 7:**
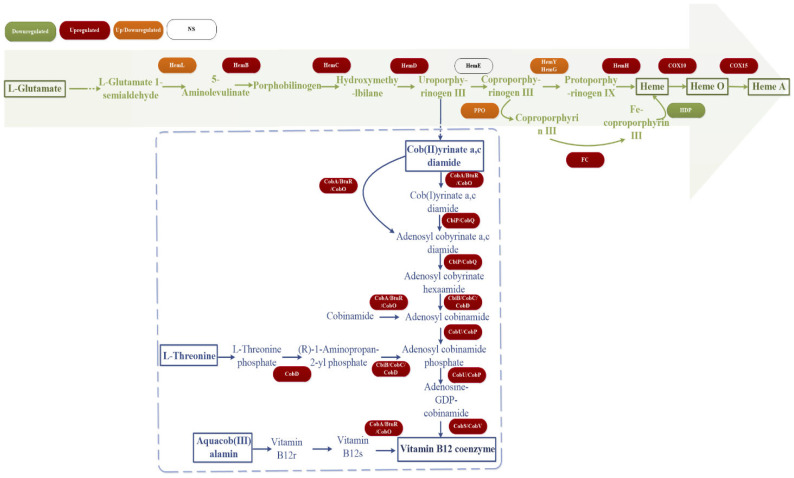
Changes in gene expression of heme and coenzyme B12 synthesis pathways.

Heme biosynthesis exhibited biphasic regulation: Core synthesis genes (*hemB/C/D/H*) were upregulated, whereas genes encoding protoporphyrinogen oxidase (*hemY/G*) and peroxidase were downregulated, reflecting dynamic iron homeostasis [63,64,65]. Comprehensive upregulation of key enzyme genes in the coenzyme B12 synthesis pathway indicates its central role as a prokaryote-specific cofactor in saline–alkali detoxification [66,67]. Perturbations in porphyrin metabolism are illustrated in Figure 7, with associated differentially expressed genes detailed in Table S5.

**Figure 8:**
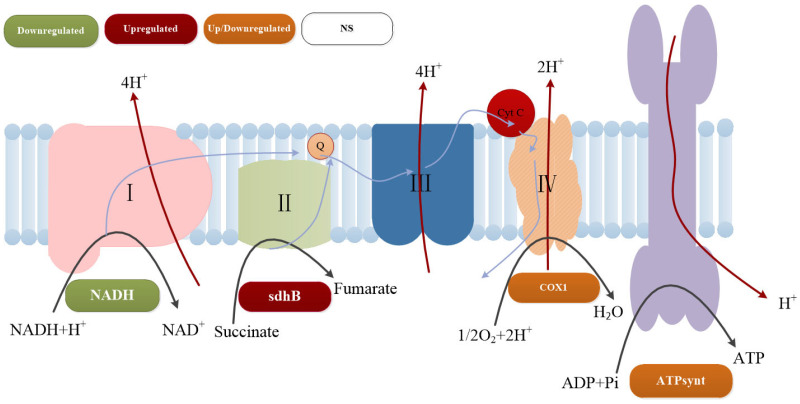
Gene expression changes in the oxidative phosphorylation pathway. (The arrows represent the transport and conversion processes of different ions in the channels of oxidative phosphorylation).

The electron transport chain associated with oxidative phosphorylation in *B. pasteurii* consists of NADH dehydrogenase (Complex I), Sdhb (Complex II), cytochrome c reductase (Complex III), and cytochrome c oxidase (Complex IV) [47]. Adaptive remodeling of oxidative phosphorylation under saline–alkali stress manifested as membrane system-specific responses in the respiratory chain. The downregulation of Complex I (NADH dehydrogenase) combined with the upregulation of Complex II (Sdhb) and Complex III (cytochrome c reductase) established an electron transport compensation mechanism. This regulatory adjustment is critical for maintaining salt–alkali tolerance, sodium ion homeostasis, and pH stability in *B. pasteurii* [68]. Significant activation of the F-type Na^+^/H^+^-translocating ATPase subunit gene *alpHa*, synergistically coordinated with upregulated inorganic pyrophosphatase, sustained transmembrane proton gradients. Conversely, downregulation of the F-type H^+^-translocating ATPase prevented proton leakage under alkaline conditions [69,70]. Metabolic reprogramming within oxidative phosphorylation pathways is graphically summarized in Figure 8, with associated differentially expressed genes systematically cataloged in Table S6.

**Figure 9:**
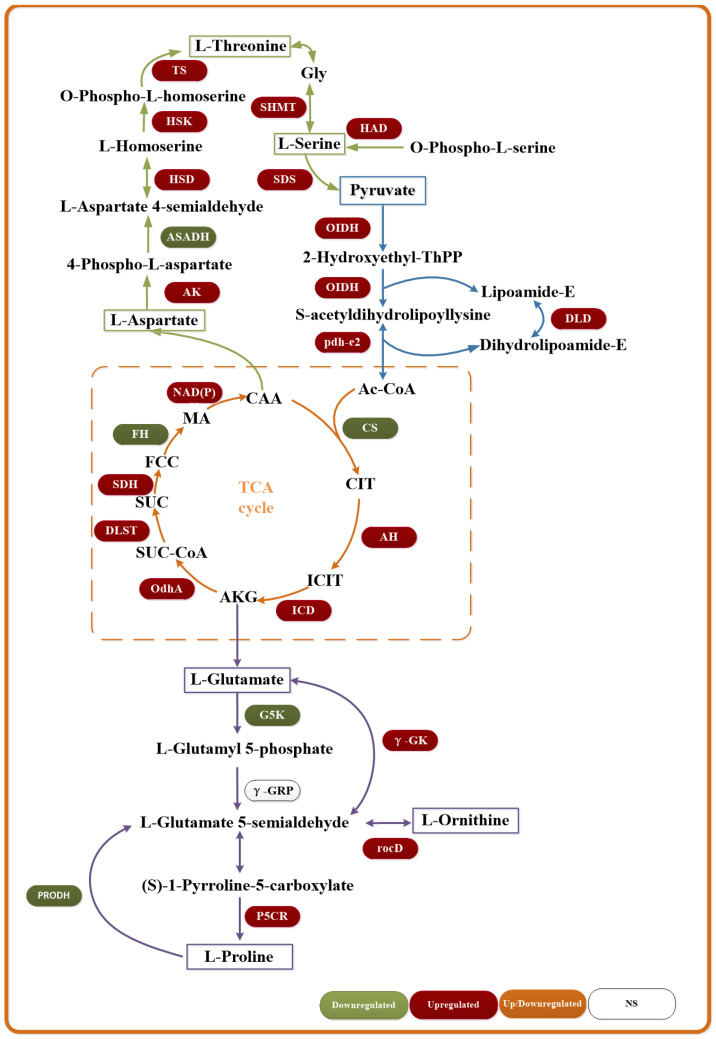
Changes in gene expression of tricarboxylic acid cycle and amino acid pathway.

The TCA cycle, a central pathway for carbohydrate, lipid, and amino acid metabolism, serves as a critical hub for bacterial energy production [71]. Compared to the original strain, the evolved strain exhibited upregulation of most TCA cycle genes under saline–alkali stress, indicating enhanced energy metabolism. Complete upregulation of Ac-CoA synthesis pathway genes suggests elevated cellular vitality in evolved strain, enabling increased ATP production to sustain cellular functions and counteract high osmolarity and elevated pH conditions. Key TCA cycle enzyme genes—pyruvate dehydrogenase and isocitrate dehydrogenase—were upregulated, driving enhanced ATP synthesis, while downregulation of citrate synthase implies metabolic flux redirection. Concurrently, the glutamate/proline biosynthesis pathway (involving ornithine aminotransferase and pyrroline dehydrogenase) was markedly activated, establishing an acidic amino acid-mediated osmoprotection system. This mechanism synergized with upregulated aspartate/serine biosynthesis genes to maintain intracellular proton homeostasis [72,73,74]. Metabolic reprogramming in the TCA cycle and amino acid pathways is visualized in Figure 9, with associated differentially expressed genes comprehensively detailed in Tables S7 and S8.

### 3.8. Comparison of Compressive Strength Repair Effect of S. pasteurii

**Figure 10:**
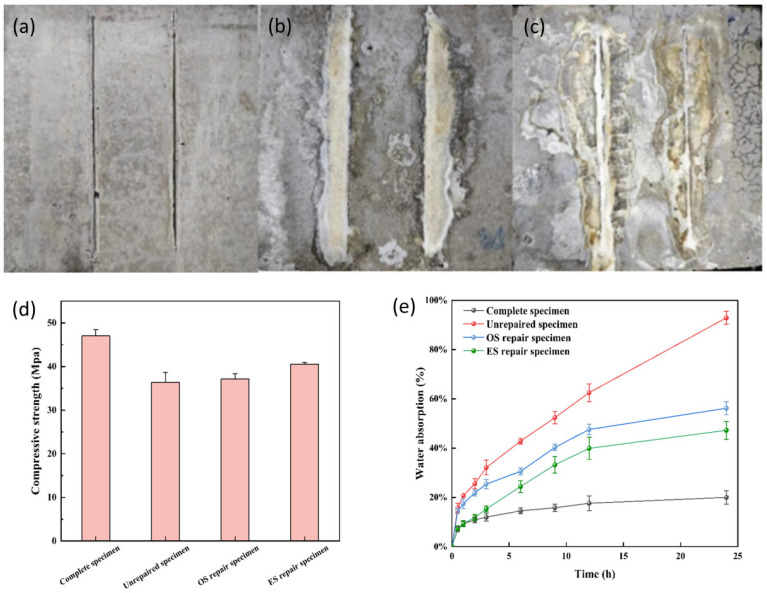
Morphological characteristics and performance evaluation of bacterial-based concrete repair: (**a**) pre-repair concrete specimen; (**b**) post-repair specimen using evolved strain; (**c**) post-repair specimen using original strain; (**d**) water absorption rate; (**e**) compressive strength variation.

The precast 0.3 mm wide microcracked concrete specimens were repaired by using the mode of urea, calcium acetate + bacterial solution, and the cracks were repaired three times a day for a total of two days. The repair results are shown in Figure 10. Figure 10a shows the crack diagram before the specimen was repaired, and Figure 10b,c shows the effect of repairing the completed cracks using the evolved strains versus the initial strains. The products of the specimens repaired with the evolved strain were better aggregated and more tightly cemented, while the products of the specimens repaired with the original strain were poorly cemented and the repair products were poorly aggregated around the crack. The fermentation broth of the evolved strain was more viscous compared to that of the original strain, which is presumed to be the result of more extracellular secretions (adhesive protein, Polysaccharide, etc.) released by the evolved strain during its growth and metabolic activities. These organic matrices were tightly adhered to the surface of the bacterium, which not only provided key nucleation sites for biomineralization, but also played a facilitating role in the process of mineral nucleation and crystal growth [75,76].

Compressive strength testing revealed the following values: intact specimens (47.05 MPa), unrepaired specimens (36.4 MPa), original strain-repaired specimens (37.15 MPa), and evolved strain-repaired specimens (40.55 MPa) (Figure 10e). Strength recovery rates reached 86.2% for evolved strain-repaired specimens and 77.3% for original strain-repaired specimens. This demonstrates the superior efficacy of evolved strains in repairing concrete cracks.

### 3.9. Comparison of the Anti Permeability Repair Effect of S. pasteurii

Post microbial restoration (MICP), water absorption of specimens treated with the original and evolved strains was evaluated alongside unrepaired and intact controls, as illustrated in Figure 10d. Water absorption increased progressively over time, stabilizing after 24 h. At 24 h, water absorption rates were 20% for intact specimens, 92.95% for unrepaired specimens, 56.22% for original strain-repaired specimens, and 47.25% for evolved strain-repaired specimens. This hierarchy (intact > evolved strain-repaired > original strain-repaired > unrepaired) confirms that evolved strain remediation significantly enhances impermeability relative to the original strain.

Evolved strains developed via adaptive strategies exhibit dual tolerance to high-salinity marine conditions and alkaline concrete environments, positioning MICP as a promising solution for marine engineering challenges. These advancements offer novel strategies for marine conservation, including mitigation of seabed liquefaction, coastal sand stabilization, and cliff erosion prevention [77].

## 4. Conclusions

Through adaptive evolution strategies, a strain of *S. pasteurii* capable of surviving in high-saline–alkali environments and exhibiting efficient mineralization was obtained. The evolved strain demonstrated growth activity and enzymatic activity under high-saline–alkali conditions comparable to the parental strain under non-stress conditions, with no significant differences in calcium carbonate yield, particle size, or crystal morphology, indicating superior biomineralization performance. Whole-genome sequencing identified five non-synonymous mutant genes, and protein structure prediction suggested that these mutations might enhance the strain’s adaptability to saline–alkali conditions. Transcriptomic analysis revealed 1082 differentially expressed genes between the evolved and parental strains, including 543 upregulated and 539 downregulated genes. Metabolic pathway analysis showed that these differentially expressed genes were primarily enriched in key pathways such as ribosome, porphyrin metabolism, oxidative phosphorylation, tricarboxylic acid (TCA) cycle, and amino acid metabolism, unveiling the molecular mechanisms underlying the evolved strain’s adaptation to high-saline–alkali environments. Notably, alterations in ribosome and oxidative phosphorylation pathways reflected adaptive adjustments in protein synthesis and energy metabolism under saline–alkali stress. In concrete repair experiments, the evolved strain demonstrated enhanced remediation efficacy, achieving an 89.3% recovery rate in compressive strength and a 48% reduction in water absorption.

This study not only deciphers the molecular basis of *S. pasteurii*’s environmental adaptation but also establishes a scalable framework for engineering microbial consortia tailored to extreme environments. Future work should explore field-scale applications in marine infrastructure and investigate the long-term durability of biomineralized repairs under cyclic tidal exposure. By integrating microbiology insights with civil engineering applications, this research advances sustainable strategies for mitigating chloride-induced degradation in marine concrete infrastructure, highlighting the potential of tailored microbial consortia for eco-friendly construction solutions.

## Figures and Tables

**Table 1:** Real-time fluoresceitative PCR primers.

Gene Name	Sequence
16s rRNA	F: AATCATTCTTGGTTCATCAAAATCACGT R: TACAATTGCTGTTTACGATGGACAC
E2C16_RS14315	F: GGCTAAAAAATCAATGGTAGCCAA R: TTACCAGCTTGCTTTTTTAACGCC
E2C16_RS11510	F: GAGCATTAAAGCTGAAGAAATCAGCGT R: GTTTTGAGCCATACCCATAACACCG
E2C16_RS9230	F: GTGAGTTCAGTTGCTCAAAAGAAAGGC R: GTTAAATAGCCCAGCACTAATTAGATCGC
E2C16_RS04705	F: TTGGATGTATCCCACCACGTGTT R: TCACGCAATTTTCCTATGCGCG
E2C16_RS14345	F: GAAATTACACGAAATGAAACCAGCTG R: CCACGTTTAGGAAGTCGTTGGAAA

**Table 2:** SNP mutated genes.

Categorization	ANNO_REGION	ANNOTATION	POS	REF	ALT	QUAL
non-synonymous mutation	ncRNA_exonic	E2C16_02500	484,733	C	T	2302.8
	ncRNA_exonic	E2C16_10645	2,200,942	G	A	2251.8
	ncRNA_exonic	E2C16_11325	2,356,286	C	A	1431.8
	ncRNA_exonic	E2C16_12620	2,635,046	T	G	1941.8
	upstream; downstream	E2C16_03190 E2C16_03185	635,957	T	A	1338.8
tautological mutation	ncRNA_exonic	E2C16_00275	62,007	C	T	2176.8
	ncRNA_exonic	E2C16_06875	1,401,963	T	G	3897.8
	ncRNA_exonic	E2C16_09620	1,965,081	G	C	1379.8
	ncRNA_exonic	E2C16_10645	2,200,938	C	A	2281.8
	ncRNA_exonic	E2C16_15700	3,242,631	G	A	1531.8
	upstream; downstream	E2C16_05300 E2C16_05305 E2C16_05295	1,095,678	A	T	1021.8

## Data Availability

The original contributions presented in this study are included in the article/Supplementary Materials. Further inquiries can be directed to the corresponding author.

## Supplementary Materials

The following supporting information can be downloaded at: https://www.mdpi.com/article/10.3390/microorganisms13071526/s1, *S. pasteurii’s* reference genome can be found in Document S1. The summary of sequencing data quality is shown in Table S1. The results of sequencing depth and coverage statistics are shown in Table S2. The predicted protein structure encoded by gene-encoding rRNA pseudouridine synthase (E2C16_02500) is shown in Figure S1. The predicted protein structure encoded by gene-encoding a GntP family permease (E2C16_10645) is shown in Figure S2. The predicted protein structure encoded by gen- encoding glycosyltransferase (E2C16_11325) is shown in Figure S3. The predicted protein structure encoded by gene-encoding a BCCT family transporter protein (E2C16_12620) is shown in Figure S4. Sequencing results of transcriptome analysis are presented in Table S3. Validation of the RNA-seq data by RT-qPCR detection is shown in Figure S5. A volcano plot of DEGs is shown in Figure S6. GO enrichment analysis is shown in Figure S7. KEGG pathway analysis is shown in Figure S8. Differentially expressed genes in ribosomal metabolic pathways are listed in Table S4. Differentially expressed genes in porphyrin metabolism are listed in Table S5. Oxidative phosphorylation pathways’ expressed genes are listed in Table S6. Metabolic reprogramming in the TCA cycle and amino acid pathways’ expressed genes are listed in Tables S7 and S8.
