# Supplementary figures and images for: Adaptive Evolution of Sporosarcina pasteurii Enhances Saline–Alkali Resistance for High-Performance Concrete Crack Repair via MICP
Source: Microorganisms. 2025 Jun 30;13(7):1526. doi: 10.3390/microorganisms13071526 (PMC12300605; doi:10.3390/microorganisms13071526)

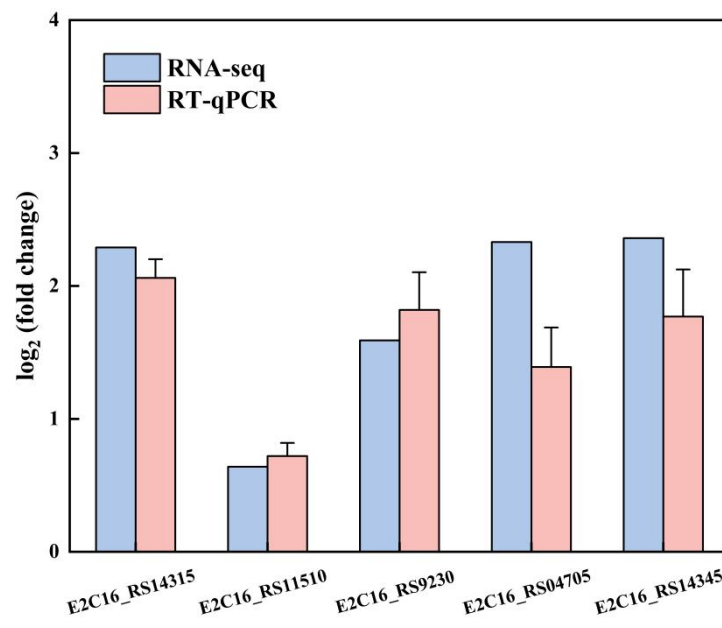

**Figure 5.** Validation of the RNA-seq data by RT-qPCR detection

Supplement: Supplementary file 1 [file microorganisms-13-01526-s001.zip › Fifure S5.pdf]

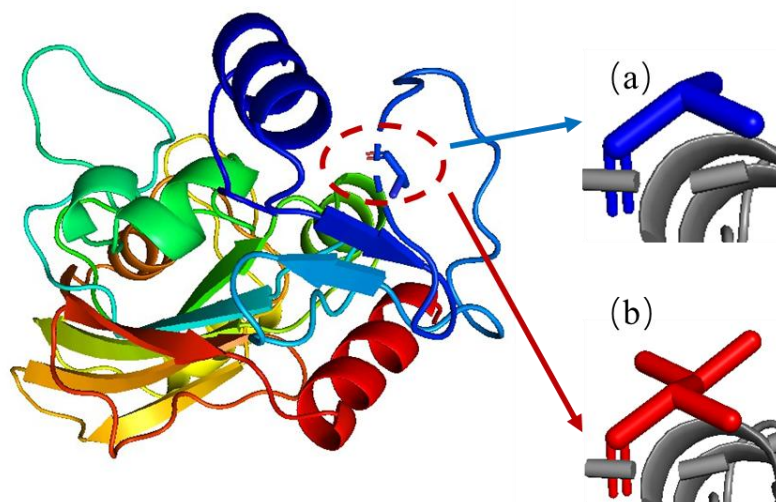

**Figure S1.** Predicted structure of the E2C16\_02500 encoding protein (a) before mutation、 (b) after mutation

Supplement: Supplementary file 1 [file microorganisms-13-01526-s001.zip › Figure S1.pdf]

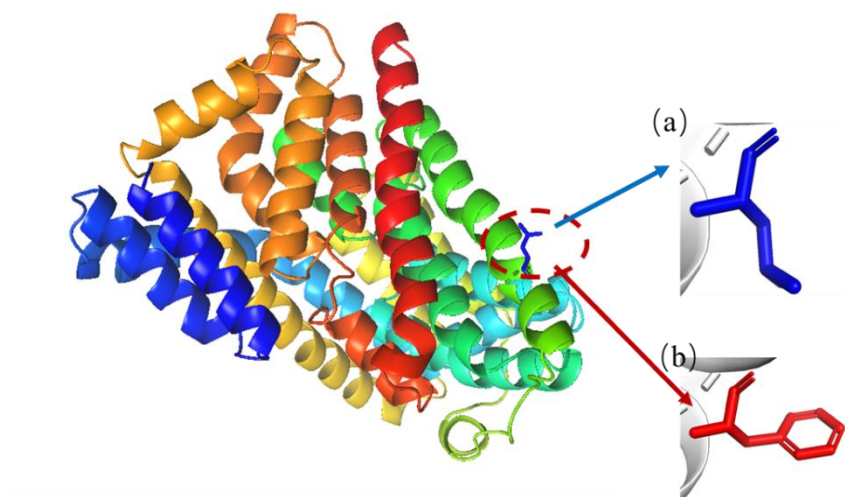

**Figure S2.** Predicted structure of E2C16\_10645 encoding protein (a) before mutation, (b) after mutation

Supplement: Supplementary file 1 [file microorganisms-13-01526-s001.zip › Figure S2.pdf]

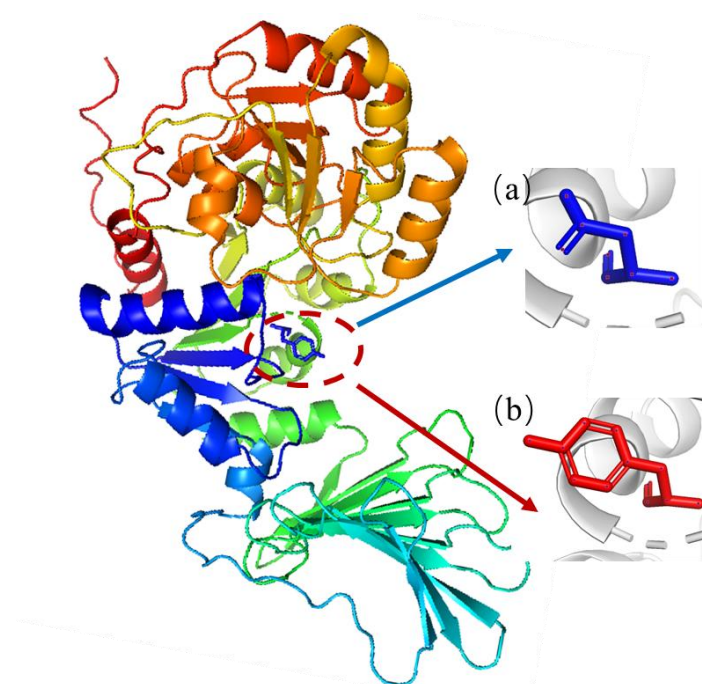

**Figure S3.** Predicted structure of the E2C16\_11325 encoding protein (a) before mutation、 (b) after mutation

Supplement: Supplementary file 1 [file microorganisms-13-01526-s001.zip › Figure S3.pdf]

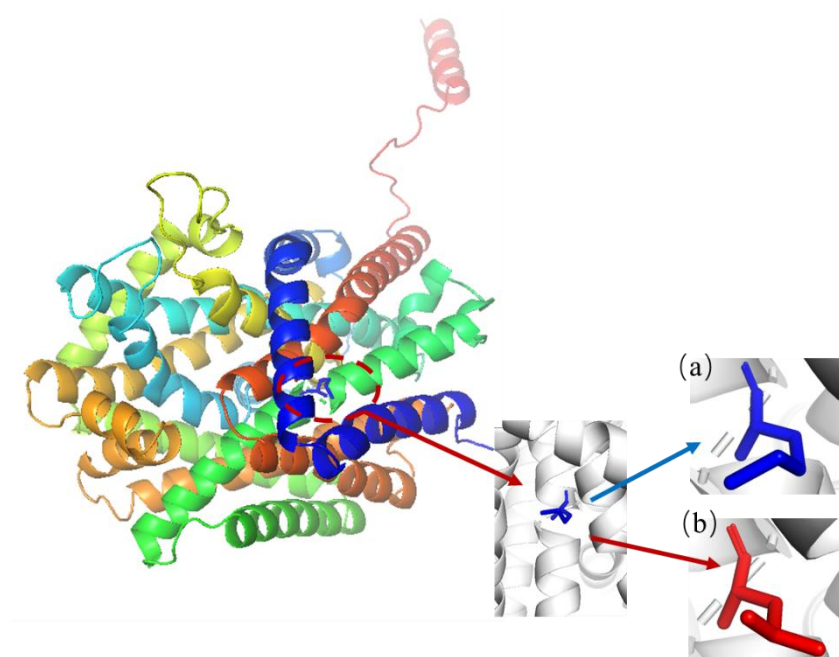

**Figure S4.** Predicted structure of the E2C16\_12620 encoding protein (a) before mutation、 (b) after mutation

Supplement: Supplementary file 1 [file microorganisms-13-01526-s001.zip › Figure S4.pdf]

# ESvsOS

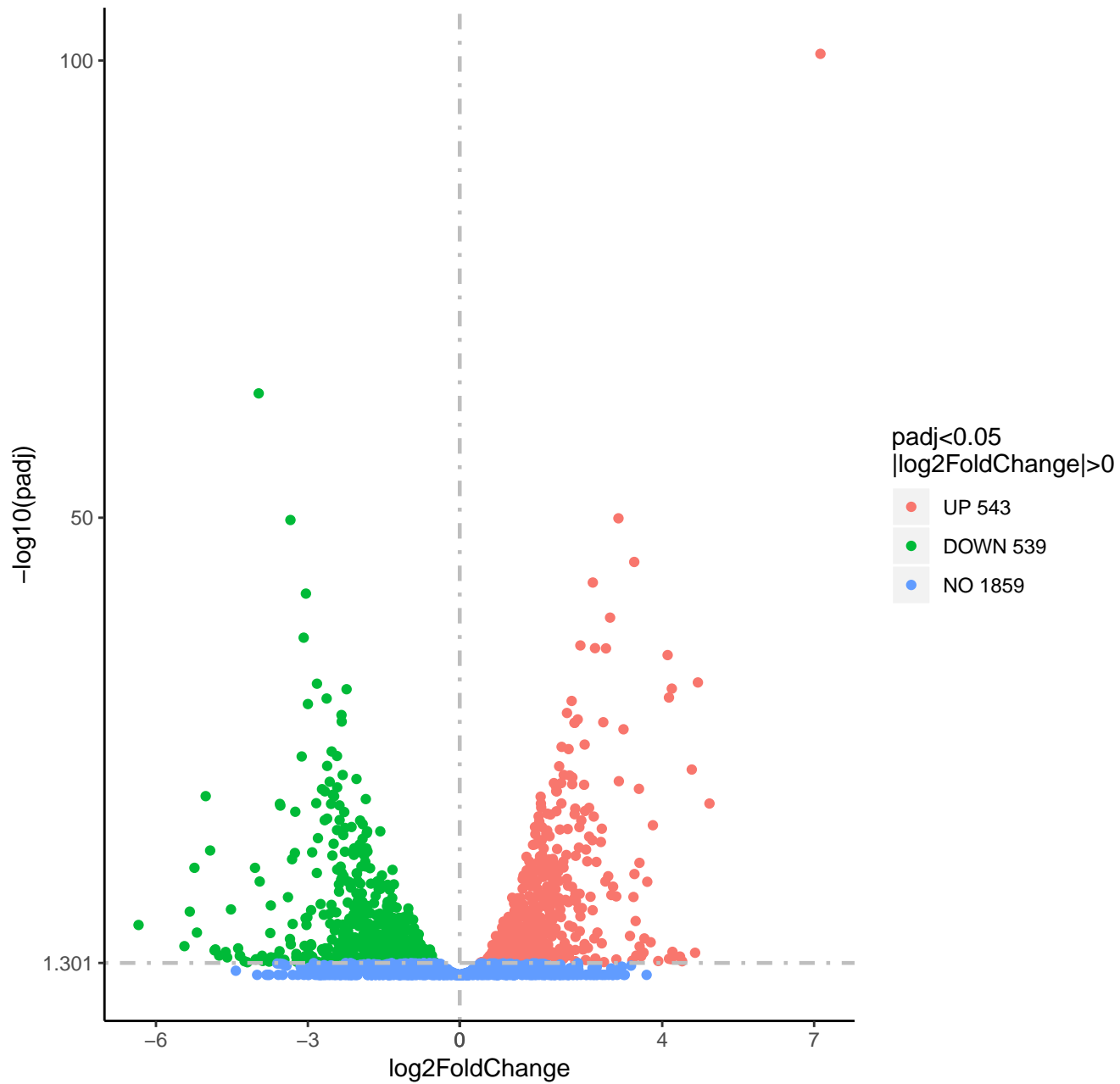

Supplement: Supplementary file 1 [file microorganisms-13-01526-s001.zip › Figure S6.pdf]

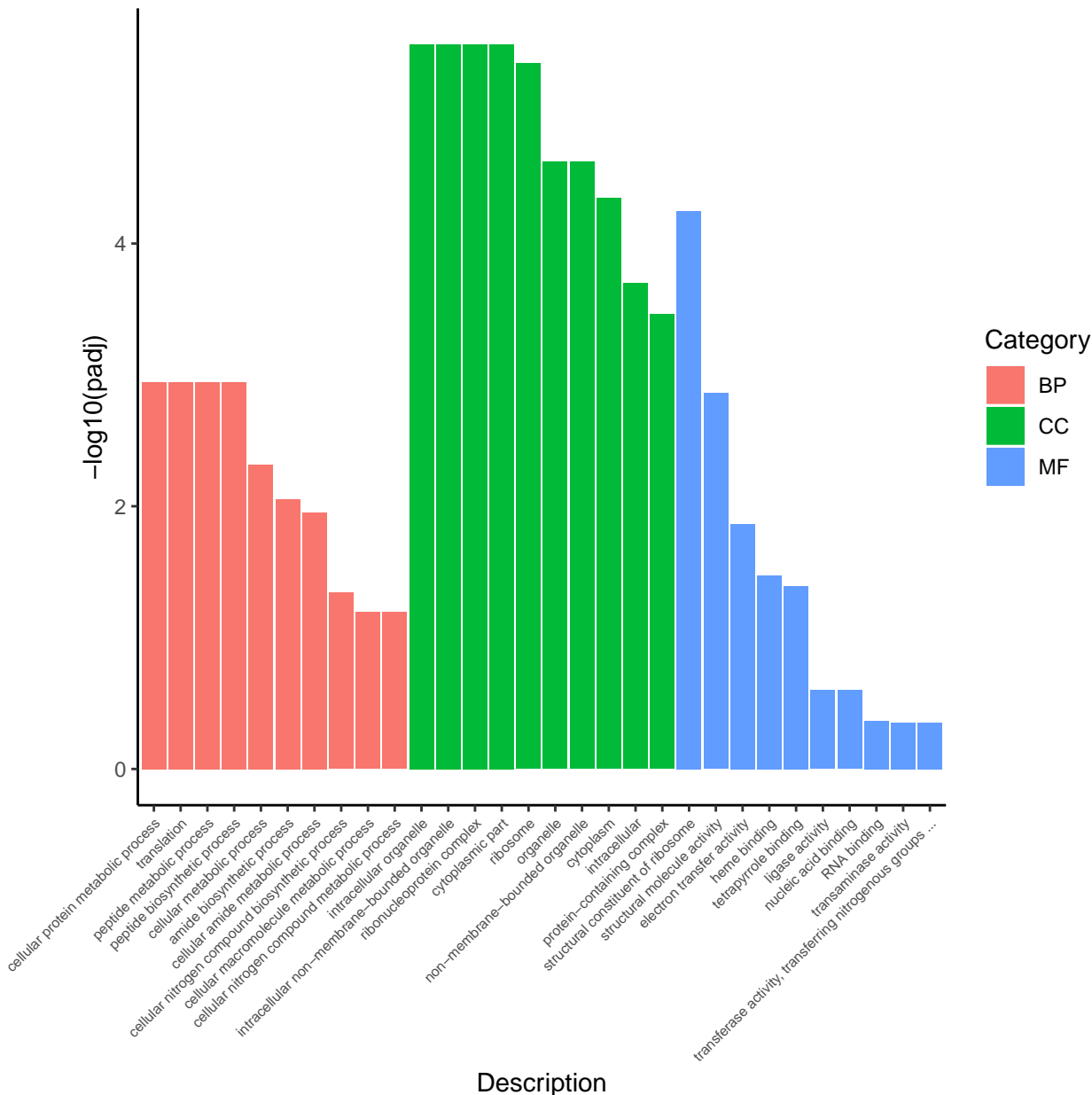

Supplement: Supplementary file 1 [file microorganisms-13-01526-s001.zip › Figure S7.pdf]

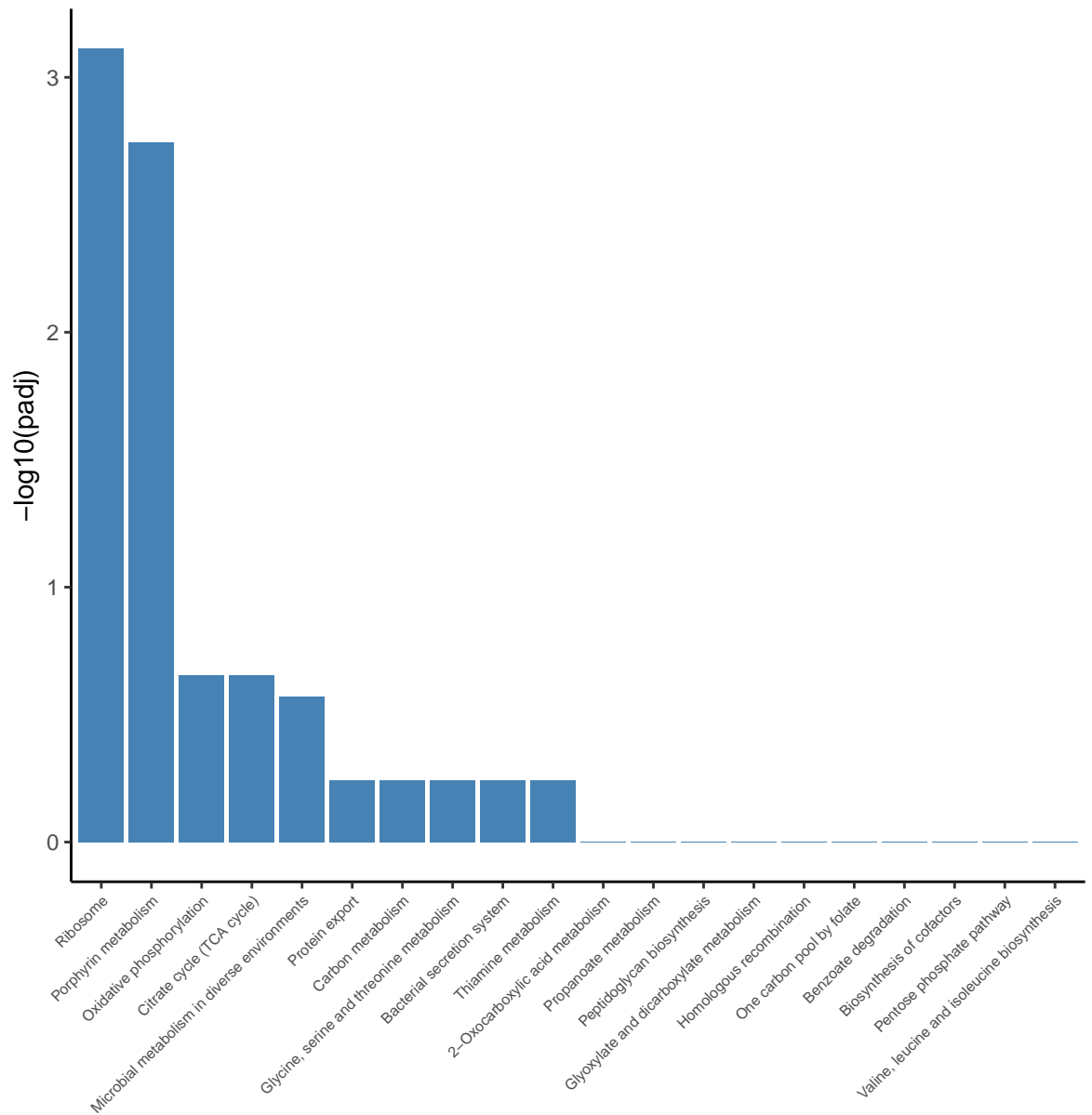

Description

Supplement: Supplementary file 1 [file microorganisms-13-01526-s001.zip › Figure S8.pdf]
